# MXenes for Solar Cells

**DOI:** 10.1007/s40820-021-00604-8

**Published:** 2021-02-21

**Authors:** Lujie Yin, Yingtao Li, Xincheng Yao, Yanzhou Wang, Lin Jia, Qiming Liu, Junshuai Li, Yali Li, Deyan He

**Affiliations:** Key Laboratory of Special Function Materials and Structure Design of the Ministry of Education, and School of Physical Science and Technology, Lanzhou University, 222 South Tianshui Road, Lanzhou, 730000 People’s Republic of China

**Keywords:** Ti_3_C_2_T_*x*_ MXene, Solar cells, Additives, Hole/electron transport layers, Electrodes

## Abstract

Application of two-dimensional MXene materials in photovoltaics has attracted increasing attention since the first report in 2018 due to their metallic electrical conductivity, high carrier mobility, excellent transparency, tunable work function and superior mechanical property. In this review, all developments and applications of the Ti_3_C_2_T_*x*_ MXene (here, it is noteworthy that there are still no reports on other MXenes’ application in photovoltaics by far) as additive, electrode and hole/electron transport layer in solar cells are detailedly summarized, and meanwhile, the problems existing in the related studies are also discussed. In view of these problems, some suggestions are given for pushing exploration of the MXenes’ application in solar cells. It is believed that this review can provide a comprehensive and deep understanding into the research status and, moreover, helps widen a new situation for the study of MXenes in photovoltaics.

## Highlights

- This review summarizes applications and developments of MXenes in solar cells by far.
- The issues needing to be addressed for performance improvement of the related solar cells are discussed.
- Suggestions are given for pushing exploration of MXenes’ application in solar cells.

## Graphical abstract

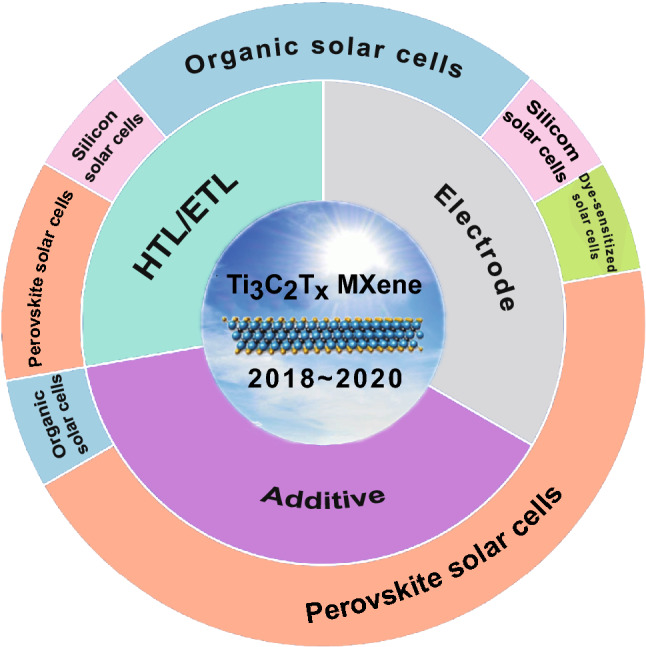

## Introduction

With the ever-increasing demand of clean and renewable energy resources [1–4], considerable attention has been devoted to the development of novel materials toward efficient solar cells [5–14]. As a family of important two-dimensional materials, MXenes, layered carbides and nitrides of transition metals first reported by the Gogotsi group in 2011 [15], which have been extensively investigated in various fields including energy storage [16–22], biomedical fields [23–25], electromagnetic applications [26–29], sensors [30–34], light-emitting diodes [35–37], water purification [38–43] and catalysis [44–47], have exhibited promising application in solar cells very recently. Among various MXenes, Ti_3_C_2_T_*x*_ (T represents some surface-terminating functional groups such as –O, –OH and –F) dominates the present study of MXenes in solar cells because of its high electrical conductivity and carrier mobility, excellent transparency and tunable work function (WF) [48–50]. Since the first report of Ti_3_C_2_T_*x*_ as an additive in the photoactive layer of MAPbI_3_ (MA: CH_3_NH_3_)-based perovskite solar cells (PSCs) in 2018 [51], its application has been extended to electrode, hole/electron transport layer (HTL/ETL), additive in HTL/ETL and the component of forming the Schottky junction-based solar cells with silicon (Si) wafers, etc.

To comprehensively understand the achievements and meanwhile to provide insights and valuable suggestions for the following development, a timely summary and discussion of the related studies is highly necessary. In this review, we first categorize the roles of Ti_3_C_2_T_*x*_ played in the reported solar cells and then follow the roles to introduce the achievements and analyze the existing problems limiting device performance improvement. Finally, a perspective to outlook the further development of the MXenes’ application in solar cells is given.

**Fig. 1:**
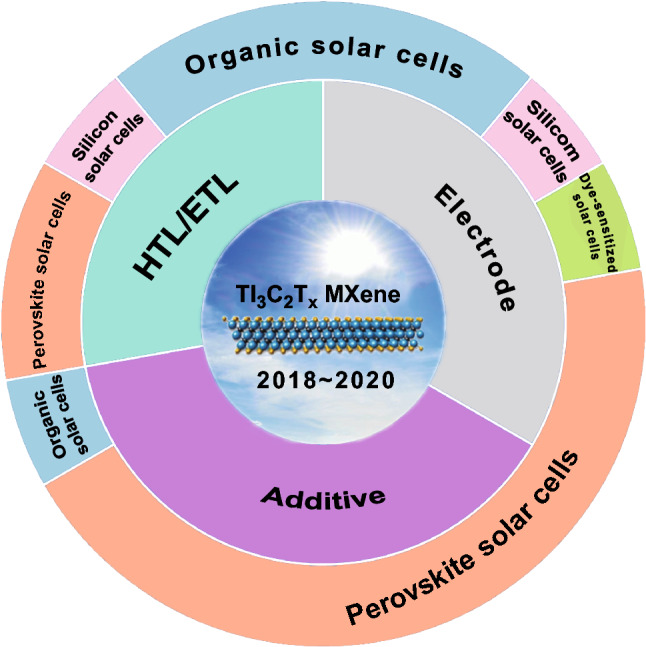
Roles of the Ti_3_C_2_T_*x*_ MXene played in application of varying solar cells. The areas correspond to the publication numbers for each application

As summarized in Fig. 1, the roles of the Ti_3_C_2_T_*x*_ MXene in application of solar cells can be categorized into three kinds, i.e., additive [51], electrode [52] and HTL/ETL [53]. In the meantime, the corresponding type of the solar cells is also summarized for each role played by Ti_3_C_2_T_*x*_. Moreover, it is noted that the corresponding areas of the roles and the solar cells in Fig. 1 are in direct proportion to the number of the reports/publications. One thus can conclude that the Ti_3_C_2_T_*x*_ MXene is mainly applied in perovskite and organic solar cells (OSCs). In the following part, the review will be extended following the role of the Ti_3_C_2_T_*x*_ MXene.

## Applications of MXenes in Solar Cells

### Additive in Perovskite Materials, ETLs/HTLs

**Fig. 2:**
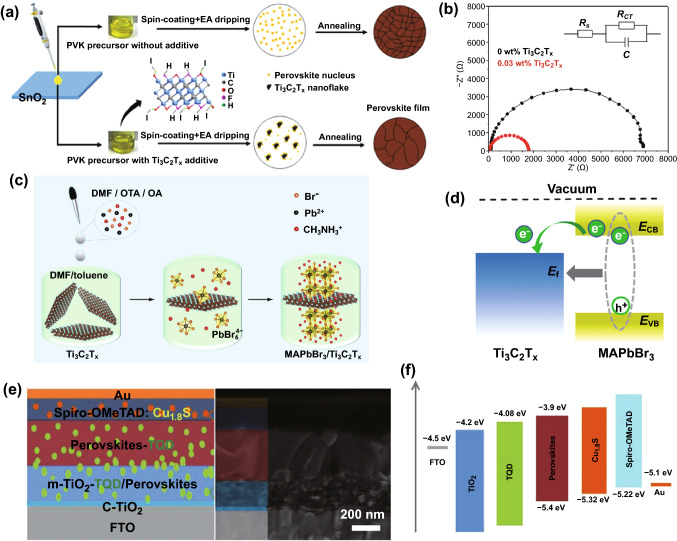
**a** Nucleation and growth routes of the MAPbI_3_-based perovskite films with and without adding the Ti_3_C_2_T_*x*_ MXene. **b** Nyquist plots of the PV devices with and without 0.03 wt% Ti_3_C_2_T_*x*_ addition measured in the dark with a bias of 0.7 V. Copyright © 2018 WILEY‐VCH Verlag GmbH & Co. KGaA, Weinheim. **c** Schematic illustration of the preparation process of surface-decorated MAPbBr_3_ nanocrystals by few-layer Ti_3_C_2_T_*x*_ MXene nanosheets, i.e., MAPbBr_3_/Ti_3_C_2_T_*x*_ heterostructures. **d** Energy-level alignment and electron transfer between the MAPbBr_3_ crystals and the coated Ti_3_C_2_T_*x*_ nanosheets. Copyright © 2020 WILEY‐VCH Verlag GmbH & Co. KGaA, Weinheim. **e** Device architecture and cross-sectional scanning electron microscopy (SEM) image, and **f** energy-level alignment of the perovskite solar cell with the embedded ultrathin Ti_3_C_2_T_*x*_ quantum dots in the perovskite layer and the ETL/TiO_2_ interface and Cu_1.8_S in the Spiro-OMeTAD HTL. Copyright © 2020 WILEY‐VCH Verlag GmbH & Co. KGaA, Weinheim

In 2018, Guo et al. first reported addition of Ti_3_C_2_T_*x*_ into the MAPbI_3_-based perovskite absorber [51], initiating exploration of the MXenes’ application in solar cells. Their study indicates that addition of Ti_3_C_2_T_*x*_ can retard the nucleation process of MAPbI_3_ (see the schematic diagram in Fig. 2a), resulting in the enlarged crystal size. Moreover, the Ti_3_C_2_T_*x*_ additive is highly beneficial to accelerate the electron transfer, like a “carrier bridge” [54–57], through the grain boundary, which is further confirmed by the lower charge transfer resistance for the Ti_3_C_2_T_*x*_-added device as indicated by the electrochemical impedance spectra exhibited in Fig. 2b. Thanks to these effects, the average power conversion efficiency (PCE) increases from 15.18% to 16.80%. (Note: all PCEs in this review were measured at AM 1.5G illumination.)

In 2019, Agresti et al. reported the WF adjustment of the MAPbI_3_ films and thus optimization of the energy-level alignment for improving the performance of the related solar cells by adding the Ti_3_C_2_T_*x*_ MXene [58]. It was found that the WF of the perovskite films could be effectively tuned from 4.72 to 4.37 eV without affecting other intrinsic electronic properties such as bandgap, the relative position of the valance band to the Fermi level and film morphology. The PCE of the Ti_3_C_2_T_*x*_-incorporated MAPbI_3_-based solar cells can be improved by 26.5% after simultaneously introducing Ti_3_C_2_T_*x*_ addition in the ETL, as compared to the control one without Ti_3_C_2_T_*x*_. Recently, Zhang et al. reported surface decoration of MAPbBr_3_ nanocrystals by few-layer Ti_3_C_2_T_*x*_ MXene nanosheets to form the perovskite/MXene heterostructure via* in* *situ* solution growth, as shown in Fig. 2c [59]. The facilitated electron injection from the MAPbBr_3_ nanocrystals to the Ti_3_C_2_T_*x*_ MXene because of the matched energy levels, as indicated in Fig. 2d, is beneficial to performance improvement for the related solar cells.

Very recently, Chen et al. first reported employment of ultrathin Ti_3_C_2_T_*x*_ quantum dots (TQDs) to engineer the CsFAMA (FA: CH(NH_2_)_2_) perovskite absorber and the perovskite/TiO_2_ ETL interface, as indicated in Fig. 2e [60]. Thanks to the improved crystallinity of the perovskite film and the matched energy-level alignment (Fig. 2f) and thus the enhanced electron extraction at the perovskite/TiO_2_ ETL interface, the solar cell delivers a remarkable hysteresis-free PCE of 20.72% compared with 18.31% for the reference device and long-time ambient and light stability. It is notable that the improved performance and stability are also partly contributed by the addition of Cu_1.8_S in the HTL facilitating the perovskite crystallinity and the increased hole extraction at the perovskite/Spiro-OMeTAD HTL interface because of the matched energy-level alignment, as indicated in Fig. 2f.

**Fig. 3:**
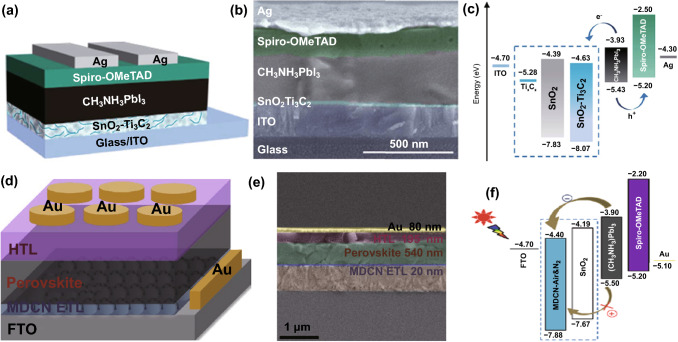
**a** Device architecture, **b** cross-sectional SEM image and **c** schematic energy-level diagram of each component for the PV device of ITO/Ti_3_C_2_T_*x*_ MXene-added SnO_2_ ETL/MAPbI_3_/Spiro-OMeTAD/Ag. Copyright © 2019 The Royal Society of Chemistry. **d** Device architecture, **e** cross-sectional SEM image and **f** schematic energy-level diagram of each component for the PSCs using MDCN as the ETL

Besides addition in the active layer, embedding the Ti_3_C_2_T_*x*_ MXene in ETLs/HTLs has also been reported. In 2019, Yang et al. reported modification of the SnO_2_ ETL by adding 1.0 wt‰ Ti_3_C_2_T_*x*_ for the MAPbI_3_-based PSCs (Fig. 3a, b for the schematized architecture and cross-sectional SEM image of the device). Thanks to the facilitated electron transport and enhanced hole blocking because of the optimized energy-level alignment due to Ti_3_C_2_T_*x*_ addition (Fig. 3c), PCE increases to 18.34% from 17.23% for the control device without Ti_3_C_2_T_*x*_ addition [61]. Huang et al. further advanced the Ti_3_C_2_T_*x*_ MXene-added SnO_2_ ETL by introducing TiO_2_ with a suitable crystal phase for forming an effective heterojunction structure, named a multi‑dimensional conductive network (MDCN) structure, as exhibited in Fig. 3d, e. Owing to the matched energy-level alignment (Fig. 3f) of the ETL with the (FAPbI_3_)_0.97_(MAPbBr_3_)_0.03_ photoactive and FTO transparent conductive layers, a PCE increment from 16.83% (SnO_2_ ETL) to 19.14% is achieved. Moreover, the MDCN-incorporated device exhibits high toleration to moisture and maintains ~ 85% of the initial performance for more than 45 days in 30–40% humidity air due to an oxygen vacancy scramble effect [62].

Besides modification of the MAPbI_3_ photoactive layer using Ti_3_C_2_T_*x*_, Agresti et al. also incorporated Ti_3_C_2_T_*x*_ into the TiO_2_ ETL to finely tune its WF, i.e., from 3.91 to 3.85 eV that benefits for tuning the interface energy-level alignment between the perovskite absorber and the TiO_2_ ETL, thus reducing the barrier height and enhancing charge transfer [58]. Based on dual addition and optimization in both the MAPbI_3_ photoactive and TiO_2_ electron transport layers, the device delivers a PCE of 20.14%, ~ 26.5% higher than that of the control device without Ti_3_C_2_T_*x*_ addition. Moreover, it was found that the Ti_3_C_2_T_*x*_ addition reduces hysteresis in the current density–voltage (*J*–*V*) curves and meanwhile enhances long-time exposure stability of the PSCs. Very recently, this group further investigated the MAPbI_3_ perovskite/Ti_3_C_2_T_*x*_-based MXene interface using density functional theory calculations, and the results show that the interface WF exhibits a strong nonlinear behavior when the relative concentrations of OH-, O- and F-terminating groups are varied, providing a deep insight regarding the energy-level alignment for high-performance device fabrication [63].

**Fig. 4:**
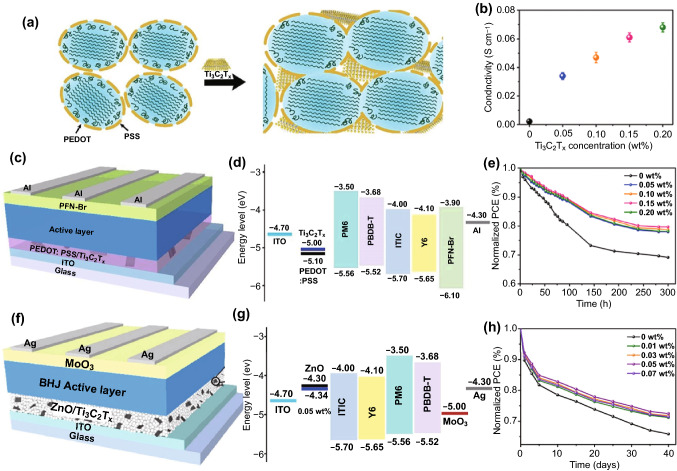
**a** Schematic illustration of morphological and structural modification in PEDOT:PSS with incorporation of the Ti_3_C_2_T_*x*_ MXene nanosheets. **b** Electrical conductivity of PEDOT:PSS with varying Ti_3_C_2_T_*x*_ additions on bare glass. **c** Device configuration and **d** energy-level diagram of each component for the OSC using Ti_3_C_2_T_*x*_-modified PEDOT:PSS as the HTL. **e** Stability test for the devices with varying Ti_3_C_2_T_*x*_ additions based on the PBDB-T:ITIC photoactive layer measured in a N_2_ glove box. Copyright © The Royal Society of Chemistry. **f** Device configuration and **g** energy-level diagram of each component for the OSC using ZnO/Ti_3_C_2_T_*x*_ as the ETL. **h** Stability test of the devices based on the PBDB-T:ITIC photoactive with varying Ti_3_C_2_T_*x*_ additions without encapsulation in air. Copyright © 2020 Elsevier B.V

Similarly, adding the Ti_3_C_2_T_*x*_ MXene into HTLs also can improve device performance. Recently, Hou et al. reported modification of the conductive polymer, PEDOT:PSS (poly(3,4-ethylenedioxythiophene):poly(styrenesulfonate)), normally as the HTL in OSCs using the Ti_3_C_2_T_*x*_ MXene nanosheets [64]. As demonstrated in Fig. 4a, after adding the MXene nanosheets, more charge transfer channels between PEDOT nanocrystals can be formed. In the meantime, the conformational transformation of PEDOT from a coil structure to a linear/expansion coil structure can be induced, hence leading to the improved electrical conductivity for the modified PEDOT:PSS, as verified by the conductivity measurement shown in Fig. 4b. Using the modified PEDOT:PSS as the HTL, the OSCs based on the non-fullerene PBDB-T:ITIC system were constructed, as schematized in Fig. 4c. Thanks to the improved electrical conductivity and matched energy-level alignment with the neighboring components (Fig. 4d) for the Ti_3_C_2_T_*x*_-modified PEDOT:PSS, a PCE of 11.02% is achieved as compared to 9.72% for the control device using pure PEDOT:PSS as the HTL. When using the PM6:Y6 system as the active layer, a PCE of 14.55% can be delivered for the case of employing the Ti_3_C_2_T_*x*_-modified PEDOT:PSS HTL as compared to 13.10% for the control device using pure PEDOT:PSS. Moreover, appropriate addition of the Ti_3_C_2_T_*x*_ MXene nanosheets can improve the performance stability, as indicated in Fig. 4e.

Moreover, they also tried to add the Ti_3_C_2_T_*x*_ MXene nanosheets in zinc oxide (ZnO) to fabricate a novel ZnO/Ti_3_C_2_T_*x*_ hybrid ETL by precisely controlling the amount of Ti_3_C_2_T_*x*_ in the sol–gel ZnO precursor solution [65]. The nanosheets act as the “electron bridges,” as aforementioned [54, 55], between the ZnO nanocrystals, thus providing additional charge transport pathways. Meanwhile, the Ti_3_C_2_T_*x*_ MXene passivates the surface of the ZnO nanocrystals by forming the Zn–O–Ti bonding, through which electrons can transfer. This ZnO/Ti_3_C_2_T_*x*_ hybrid ETL with excellent optical and electrical properties was applied in fullerene (PBDB-T:ITIC and PM6:Y6) and non-fullerene (PTB7:PC_71_BM) OSCs (Fig. 4f, g for the architecture and energy-level alignment of the device), and the improved PCEs of 12.20%, 16.51% and 9.36% from 10.56%, 14.99% and 8.18% for the control devices using the pristine ZnO ETLs were achieved by the solar cells based on the PBDB-T:ITIC, PM6:Y6 and PTB7:PC_71_BM photoactive layers, respectively. Moreover, the improved stability for the devices using the ZnO/Ti_3_C_2_T_*x*_ hybrid ETLs was observed, as shown in Fig. 4h.

### Electrode

The newly reported electrical conductivity of the Ti_3_C_2_T_*x*_ MXene has reached as high as 15,100 S cm^–1^ [66], and moreover, high transparency, outstanding flexibility and adjustable WF are associated with it [67–69]. All these properties make Ti_3_C_2_T_*x*_ suitable as electrodes in optoelectronic devices including solar cells. In the following, the review will be expanded in the sequence of the perovskite-based, organic, Si wafer-based and dye-sensitized solar cells.

**Fig. 5:**
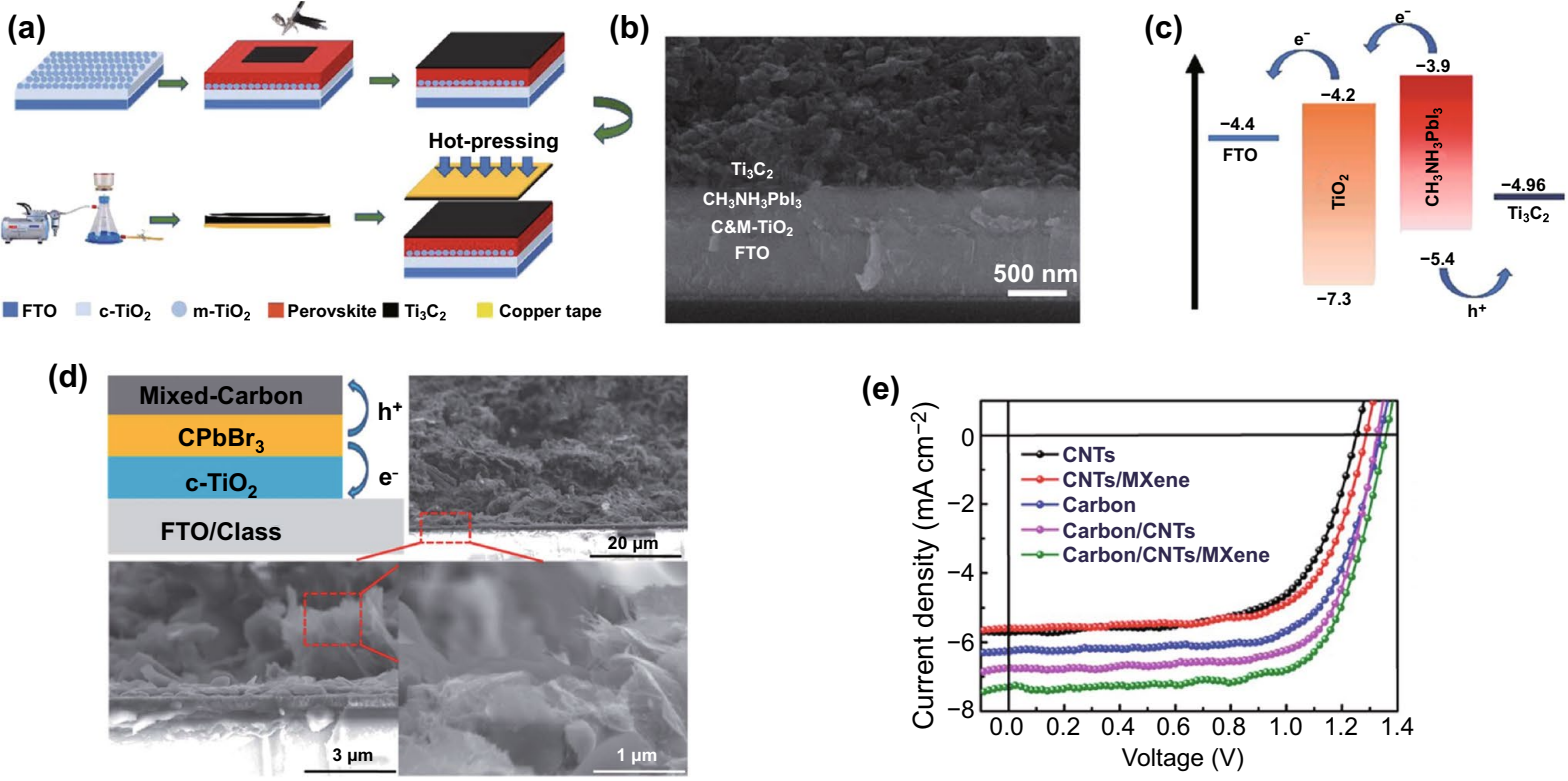
**a** Fabrication process of the MAPbI_3_-based PSCs with a Ti_3_C_2_T_*x*_ back electrode prepared using a hot-pressing method. **b** Cross-sectional SEM image and **c** energy-level alignment of each component for the MAPbI_3_-based PSCs. Copyright © 2019 The Royal Society of Chemistry. **d** Device configuration of the CsPbBr_3_-based solar cells using the mixed carbon electrode and cross-sectional SEM images of the mixed carbon electrode. **e** Illuminated *J*–*V* curves of the solar cells with different types of electrodes. Copyright © 2020 The Royal Society of Chemistry

In 2019, Gao et al. reported use of Ti_3_C_2_T_*x*_ MXene materials as the back electrode in noble-metal-free MAPbI_3_-based PSCs through a simple hot-pressing method, and Fig. 5a–c shows the preparation procedure, cross-sectional SEM image and energy-level alignment of each component for the devices [52]. One notes that as the back electrode, the Ti_3_C_2_T_*x*_ MXene can facilitate hole injection from the MAPbI_3_ photoactive layer, and the device delivers a PCE of up to 13.83%, ~ 27.2% higher than that of the control device using the carbon electrode. Moreover, thanks to the seamless interfacial contact, the device exhibits improved stability compared with the control one. Recently, Mi et al. employed a mixed electrode consisting of carbon, carbon nanotube (CNT) and Ti_3_C_2_T_*x*_ in CsPbBr_3_-based PSCs, and Fig. 5d shows the device architecture and the cross-sectional SEM images of the mixed electrode. As indicated in Fig. 5e, the devices using the Ti_3_C_2_T_*x*_-incorporated electrodes exhibit improved performance compared with the devices employing the electrodes without Ti_3_C_2_T_*x*_ [70].

**Fig. 6:**
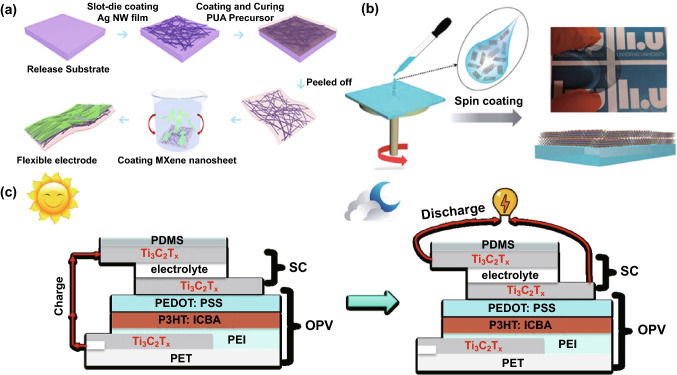
**a** Fabrication process of the flexible transparent electrodes based on Ti_3_C_2_T_*x*_ MXene nanosheets and Ag nanowire networks. Copyright © 2019 American Chemical Society. **b** Schematic of the preparation (left), optical photograph (upper right) and cross-sectional demonstration (lower right) of the Ti_3_C_2_T_*x*_ transparent flexible electrode for the PV supercapacitors. **c** Working principle of the semitransparent, flexible PV supercapacitor in the charge (left) and discharge states. Copyright © 2020 The Royal Society of Chemistry

In 2019, Tang et al. combined Ti_3_C_2_T_*x*_ nanosheets with Ag nanowire networks to fabricate the transparent, highly conductive and flexible hybrid electrodes for flexible OSCs that are based on different combinations of organic active materials including PTB7-Th:PC_71_BM, PBDB-T:ITIC and PBDB-T:ITIC:PC_71_BM [71]. This MXene/Ag nanowire hybrid electrode, prepared via a simple and scalable solution-processed method as shown in Fig. 6a, exhibits excellent performance. The flexible ternary (PBDB-T:ITIC:PC_71_BM) OSCs using this hybrid transparent electrode deliver a champion PCE of 8.30% and meanwhile exhibit robust mechanical performance, i.e., 84.6% retention of the initial PCE after 1000 bending and unbending cycles to a 5-mm bending radius.

Recently, Qin et al. reported utilization of the Ti_3_C_2_T_*x*_ MXene thin film as a common electrode to fabricate the MXene-based all-solution-processed semitransparent flexible photovoltaic (PV) supercapacitor by integrating a flexible OSC and a transparent MXene supercapacitor in the vertical direction [72]. Figure 6b shows the schematic of the preparation (left), optical photograph (upper right) and cross-sectional demonstration (lower right) of the transparent flexible electrode, and the device configuration and working principle are shown in Fig. 6c. The flexible OSCs with Ti_3_C_2_T_*x*_ as the transparent electrode can deliver a high PCE of 13.6%, comparable with that of the control device using an ITO electrode. The PV supercapacitor exhibits an average visible transmission of 33.5% and the maximum storage efficiency and overall efficiency of up to 88% and 2.2%, respectively. In addition, this strategy is suitable for blading, printing and roll-to-roll manufacturing, which is promising for the production of cost-efficient flexible PV supercapacitors to satisfy the increasing energy demands for portable, wearable and miniature electronic devices.

**Fig. 7:**
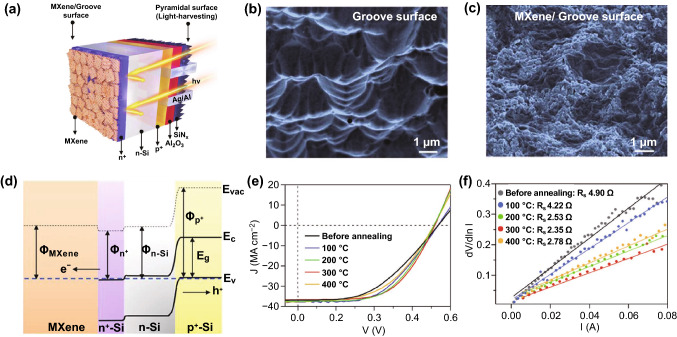
**a** Schematic illustration for the *n*^+^–*p*–*p*^+^ Si solar cell using the Ti_3_C_2_T_*x*_ MXene as the electrode contacted with the *n*^+^ emitter. SEM images of grooves on the *n*^+^ side surface **b** before and **c** after MXene coating. **d** Energy-level alignment. *Φ* is a work function; *E*_g_, *E*_c_ and *E*_v_ are the energy bandgap, conduction band and valence band of Si. **e** Illuminated *J*-*V* curves before and after 30 s RTA treatment at varying temperatures. **f** Series resistance values deduced from the *J*–*V* measurement for the samples before and after the RTA process. Copyright © 2019 WILEY‐VCH Verlag GmbH & Co. KGaA, Weinheim

In 2019, Fu et al. drop-casted the Ti_3_C_2_T_*x*_ MXene solution on the groove surface of the *n*^+^-Si emitter as the back electrode in an *n*^+^–*n*–*p*^+^ Si solar cell (see Fig. 7a–c for the device architecture, groove surface before and after MXene coating) [73]. The ohmic contact between Ti_3_C_2_T_*x*_ and *n*^+^-Si (see Fig. 7d for the energy-level alignment) facilitates electron transfer from the *n*^+^-Si emitter and thus suppresses charge carrier recombination, resulting in good output of the short-circuit current density (*J*_sc_) and open-circuit voltage (*V*_oc_). Moreover, it was found that the rapid thermal annealing (RTA) treatment of 30 s can further improve the electrical contact and physical adhesion between the MXene coating and the *n*^+^-Si substrate, leading to the reduced series resistance (it can be concluded from the increased slope for the *J*–*V* curves around *V*_oc_ in Fig. 7e and also verified by the resistance measurement, as exhibited in Fig. 7f) and thus a remarkable improvement of PCE to 11.5%. In some cases, MXenes serve both the electrode and the component of forming the Schottky junction with Si. For example, Yu et al. reported a Schottky junction solar cell fabricated based on Ti_3_C_2_T_*x*_ and *n*-Si, where the Ti_3_C_2_T_*x*_ electrode meanwhile serves as a transparent conducting film for charge collection, as shown in Fig. 10a (later) [74]. For this content, we would like to more detailedly discuss in the following part of MXenes as HTLs/ETLs.

The Ti_3_C_2_T_*x*_ MXene was also employed to fabricate the counter electrode (CE) in quantum dot-sensitized solar cells (QDSCs). In 2019, Chen et al. reported a composite CE consisting of hydrothermally grown CuSe nanoparticles on Ti_3_C_2_T_*x*_ MXene nanosheets that were screen-printed onto a graphite sheet [75]. This composite CE possesses the better electrical conductivity for electron transfer and a larger specific surface area to provide more active sites for polysulfide electrolyte reduction, as compared to CuSe- and Ti_3_C_2_T_*x*_-based CEs, respectively. The PCE of 5.12% can be achieved by the device using the CuSe/Ti_3_C_2_T_*x*_ composite CE with an optimal mass ratio. As a comparison, the devices using the CuSe- and Ti_3_C_2_T_*x*_-based CEs deliver the PCE of 3.47% and 2.04%, respectively. Similarly, Tian et al. fabricated the CuS/Ti_3_C_2_ composite CEs via a facile ion-exchange method at room temperature, exhibiting a significantly faster electrocatalytic rate toward the polysulfide reduction than pure CuS [76]. The QDSC based on this composite CE delivers an overall PCE of 5.11%, which is 1.5 times obtained from the device using the pure CuS CE. The performance enhancement is mainly attributed to the combined advantages of the excellent conductivity of the Ti_3_C_2_ skeleton and the abundant catalytically active sites of the CuS nanoparticles.

### HTL/ETL

**Fig. 8:**
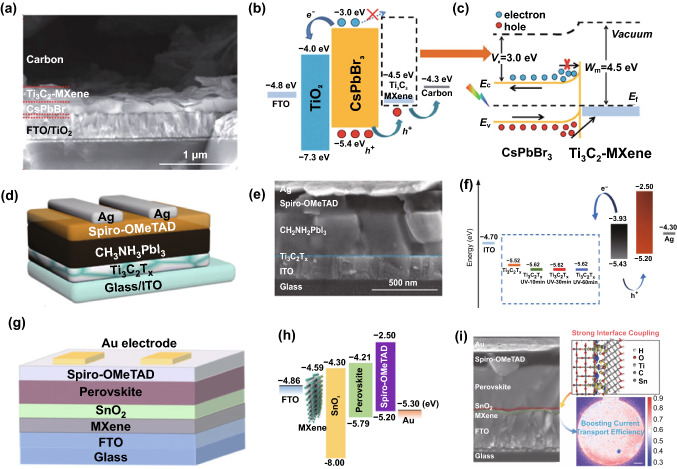
CsPbBr_3_-based PSCs using the Ti_3_C_2_T_*x*_ nanosheet layer as the HTL: **a** Cross-sectional SEM image, **b** energy-level alignment and **c** carrier transport mechanism at illumination. Copyright © 2019 The Royal Society of Chemistry. **d** Device architecture and **e** cross-sectional SEM image, **f** energy-level alignment of each layer. Copyright © 2019 WILEY‐VCH Verlag GmbH & Co. KGaA, Weinheim. **g** Device configuration and **h** energy-level alignment of the device. **i** Cross-sectional SEM image of the device (left), first-principle optimized structures and electron density differences for SnO_2_/Ti_3_C_2_(OH)_2_ (upper right), and spatially resolved mapping of the current transport efficiency of the MXene-inserted device (lower right). Copyright © 2020 American Chemical Society

Owing to the easily tunable WF, the Ti_3_C_2_T_*x*_ MXene can also be applied as an HTL or ETL, and the related reports in PSCs, OSCs and crystalline Si solar cells can be found. In 2019, Chen et al. reported insertion of Ti_3_C_2_T_*x*_ nanosheets between the CsPbBr_3_ active layer and the carbon electrode as the HTL [77], as indicated in Fig. 8a. The electron potential barrier because of the inserted Ti_3_C_2_T_*x*_ layer (Fig. 8b, c) effectively blocks the transfer of electrons from CsPbBr_3_ to the carbon electrode and thus mitigates the electron–hole recombination. In addition, the CsPbBr_3_ grains can be well passivated by the functional groups in Ti_3_C_2_T_*x*_, thus reducing the trap defects in the CsPbBr_3_ film and improving the perovskite film quality. A high initial PCE of 9.01% is obtained for the PSCs with long-term stability for more than 1900 h in a moisture environment and over 600 h under thermal conditions.

In the same year, employing the Ti_3_C_2_T_*x*_ layer as a novel ETL for MAPbI_3_-based PSCs was reported by Yang et al. [78], and Fig. 8d, e shows the device architecture and cross-sectional SEM image of the solar cell. Under the assistance of ultraviolet–ozone (UV–O_3_) treatment, the Fermi level of Ti_3_C_2_T_*x*_ could be downshifted from − 5.52 to − 5.62 eV, as indicated in Fig. 8f, and meanwhile, the interface properties between Ti_3_C_2_T_*x*_ and MAPbI_3_ were improved because of the formation of the additional oxide-like Ti–O bonds on the surface of Ti_3_C_2_T_*x*_ (Ti_3_C_2_O_2_). Thanks to the enhanced hole blocking because of the downshifted Fermi level of Ti_3_C_2_T_*x*_ and meanwhile to the improved MAPbI_3_/Ti_3_C_2_T_*x*_ interface, a champion PCE of 17.17% was thus achieved for the device employing the Ti_3_C_2_T_*x*_ MXene film with 30 min UV–O_3_ treatment. However, the device using Ti_3_C_2_T_*x*_ without UV–O_3_ treatment only delivers the 5.00% PCE. Further investigations verified that the charge transfer was truly enhanced for the case with the optimal UV–O_3_ treatment of 30 min, as indicated by the EIS measurement. In addition, UV–O_3_ treatment to Ti_3_C_2_T_*x*_ also contributes to improve the device stability.

Recently, Wang et al. reported a perovskite solar cell with a thin Ti_3_C_2_T_*x*_ MXene layer inserted between the F-doped SnO_2_ (FTO) electrode and the SnO_2_ ETL [79], and Fig. 8g, i shows the architecture and cross-sectional SEM image of the device. As indicated in Fig. 8h, the inserted MXene thin layer is favorable to form matched energy-level alignment between FTO and the SnO_2_ ETL, thus facilitating electron transport from SnO_2_ to FTO. Meanwhile, the strong interaction and electron hybridization between MXene and SnO_2_ can be introduced (see Fig. 8i for the simulated structure), thus leading to the enhanced electron mobility in SnO_2_. Moreover, the surface of the SnO_2_ ETL becomes more hydrophobic and smoother than the case without MXene, which is beneficial for growing high-quality perovskite layers. It was also found that compared with the case without MXene, non-radiative recombinations were significantly suppressed by the MXene-modified SnO_2_ ETL together with the remarkably improved homogeneity and reduced carrier transport loss (Fig. 8i). Thanks to these synergetic effects introduced by the MXene thin layer, the related device delivers a stabilized PCE of 20.65% (< 19.00% for the control device without MXene) with an ultralow saturated current density and negligible hysteresis.

**Fig. 9:**
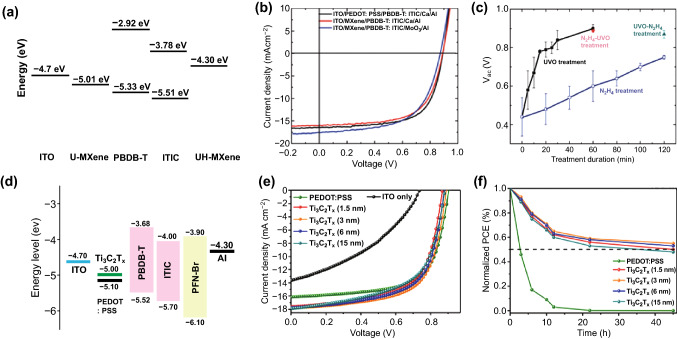
PBDB-T:ITIC-based OSCs using the UV–O_3_ and/or N_2_H_4_-treated Ti_3_C_2_T_*x*_ MXene as the ETL/HTL: **a** Energy levels of the main components and **b** illuminated *J*–*V* curves of the PBDB-T:ITIC-based OSCs. Here, U-MXene and UH-MXene denote the MXene treated only by UV–O_3_, and first by UV–O_3_ and then by N_2_H_4_, respectively. Moreover, U-MXene is used for the hole collection in the normal OSCs, and the UH-MXene is for the electron collection in the inverted OSCs; and **c** *V*_oc_ versus the treatment duration. Copyright © 2019 The Royal Society of Chemistry. PBDB-T:ITIC-based OSCs using Ti_3_C_2_T_*x*_ nanosheets as the HTL: **d** Energy-level alignment of each component, **e** illuminated *J*–*V* curves, and **f** stability test under the atmosphere condition without any encapsulations. Copyright © 2019 The Royal Society of Chemistry

In 2019, Yu et al. reported utilization of the UV–O_3_ treatment or N_2_H_4_ treatment to increase or decrease the WF of Ti_3_C_2_T_*x*_ (in a range between 4.08 and 4.95 eV) because of the oxidation or reduction of the C element, respectively [53]. As shown in Fig. 9a, the Ti_3_C_2_T_*x*_ MXenes with different WFs can be used as either the HTLs or ETLs for the OSCs employing PBDB-T:ITIC as the photoactive layer. The PCEs of 9.06% and 9.02% were obtained for the cases using Ti_3_C_2_T_*x*_ as electron and hole-collection buffer layers, respectively (Fig. 9b). Moreover, it was found that *V*_oc_ increases with the treatment duration, as exhibited in Fig. 9c. In the same year, Hou et al. also reported employment of Ti_3_C_2_T_*x*_ as the HTLs in PBDB-T:ITIC-based OSCs to facilitate hole transport and collection, benefiting from the outstanding metallic conductivity of Ti_3_C_2_T_*x*_, improved interface contact and matched energy-level alignment as exhibited in Fig. 9d [80]. It is notable that evident enhancement of PCE can be achieved for the devices using Ti_3_C_2_T_*x*_ as the HTLs compared with the control one only using ITO (PCE: 4.21%). Moreover, the optimal Ti_3_C_2_T_*x*_-based device also outperforms the state-of-the-art PEDOT:PSS-based device, i.e., 10.53% vs. 10.11% (see Fig. 9e for the device performance comparison). Meanwhile, the Ti_3_C_2_T_*x*_-based devices also exhibit the improved long-term stability under the atmosphere condition without any encapsulations, as indicated in Fig. 9f.

**Fig. 10:**
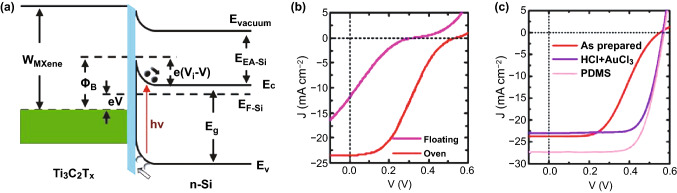
Ti_3_C_2_T_*x*_ MXene/*n*-Si solar cells: **a** Energy-level alignment of the main components (the blue strip indicates the SiO_2_ thin layer), **b** illuminated *J*–*V* curves for the devices fabricated via floating and oven transfer methods and **c** illuminated *J*–*V* curves for the devices fabricated by the oven transfer method and after the two-step (HCl + AuCl_3_) chemical treatment and further coating the PDMS antireflection film. Copyright © 2019 WILEY‐VCH Verlag GmbH & Co. KGaA, Weinheim

As briefly aforementioned, in 2019, Yu et al. reported a novel solar cell formed by depositing the Ti_3_C_2_T_*x*_ MXene on *n*-Si, where Ti_3_C_2_T_*x*_ serves as both the electrode for hole collection and the component to form the Schottky junction with *n*-Si [74], as demonstrated in Fig. 10a. Figure 10b shows the illuminated *J*–*V* curves for the devices prepared by depositing MXene using floating and oven transfer methods, respectively, with the initial efficiencies of 0.58% and 4.20%. Moreover, as exhibited in Fig. 10c, the PCE of the as-prepared device by oven transfer can be further improved, i.e., > 9% by a two-step chemical treatment using HCl and AuCl_3_ in sequence and > 10% by further introducing a PDMS antireflection layer. More investigations indicate that the SiO_2_ thin layer formed between Ti_3_C_2_T_*x*_ and *n*-Si during oven transfer plays the key role to suppress carrier recombinations and thus to achieve the higher device performance as compared to the floating method. For the improved device performance by the two-step chemical treatment, it can be attributed to the increased conductivity for the MXene layer due to the doping effect introduced by HCl, the increased Schottky barrier height (note: The WF of the MXene layer increases from 4.80 to 4.84 and further to 4.93 eV for the pristine, HCl- and AuCl_3_-treated samples) and enhanced charge transfer because of the formed Au nanoparticles from AuCl_3_.

## Conclusion and Prospect

In this review, all applications and developments of the Ti_3_C_2_T_*x*_ MXene in solar cells since the first report in 2018 are detailedly summarized. As can be seen, the Ti_3_C_2_T_*x*_ MXene mainly plays three roles, i.e., additive, electrode and charge (electron or hole) transport layer, and the type of the applied solar cells includes perovskite (mainly), organic (mainly), silicon wafer-based and quantum dot-sensitized solar cells. (Note: The functions in different roles for MXenes applied in solar cells are briefly summarized in Table 1, and meanwhile, the key device parameters are compiled in Tables 2, 3 and 4, according to the roles played by the Ti_3_C_2_T_*x*_ MXene.) Moreover, there is one report regarding a hybrid device combining electricity generation and storage, i.e., the so-called PV supercapacitor in which all electrodes are Ti_3_C_2_T_*x*_, and the organic PV device and supercapacitor share one common electrode [72].

**Table 1:** Summary of the functions/properties of MXenes in different roles played in solar cells

Additive	Accelerating the electron transfer, like an “electron” bridge Improving crystallinity of the perovskite materials Tuning the work function of the carrier transport materials and other properties such as conductivity Passivating the surface and engineering interface
Electrode	Metallic conductivity, high transparency, outstanding flexibility and adjustable work functions To form hybrid electrodes with other conducting nanomaterials, such as carbon nanotubes or metallic nanowires
HTL/ETL	Easily tunable work functions and carrier conducting properties

**Table 2:** Summary of the key parameters for the solar cells employing MXenes as an additive

Device structure	*J*_sc_ (mA cm^−2^)	*V*_oc_ (V)	FF (%)	PCE (%)	Year	References
ITO/SnO_2_/MAPbI_3_:Ti_3_C_2_T_*x*_/Spiro-OMeTAD/Au	22.26	1.03	76.00	17.41	2018	[51]
FTO/c-TiO_2_/m-TiO_2_/CsFAMA-TQD/Spiro-OMeTAD:Cu_1.8_S/Au	24.12	1.14	78.70	21.64	2020	[60]
ITO/SnO_2_-Ti_3_C_2_/MAPbI_3_/Spiro-OMeTAD/Ag	23.14	1.06	75.00	18.34	2019	[61]
FTO/TiO_2_:SnO_2_:Ti_3_C_2_T_*x*_/(FAPbI_3_)_0.97_ (MAPbBr_3_)_0.03_/Spiro-OMeTAD/Au	22.03	1.10	77.78	18.90	2020	[62]
FTO/c-TiO_2_:Ti_3_C_2_T_*x*_/m-TiO_2_:Ti_3_C_2_T_*x*_/Ti_3_C_2_T_*x*_/MAPbI_3_:Ti_3_C_2_T_*x*_/Spiro-OMeTAD/Au	23.82	1.09	77.60	20.14	2019	[58]
ITO/PEDOT:PSS:Ti_3_C_2_T_*x*_/PBDB-T:ITIC/PFN-Br/Al	17.08	0.91	70.93	11.02	2020	[64]
ITO/PEDOT:PSS:Ti_3_C_2_T_*x*_/PM6:Y6/PFN-Br/Al	25.63	0.83	68.40	14.55	2020	[64]
ITO/ZnO:Ti_3_C_2_T_*x*_/PBDB-T:ITIC/MoO_3_/Ag	18.63	0.93	70.39	12.20	2020	[65]
ITO/ZnO:Ti_3_C_2_T_*x*_/PM6:Y6/MoO_3_/Ag	26.38	0.83	75.40	16.51	2020	[65]
ITO/ZnO:Ti_3_C_2_T_*x*_/PTB7:PC_71_BM /MoO_3_/Ag	17.53	0.77	69.33	9.36	2020	[65]

**Table 3:** Summary of the key parameters for the solar cells employing MXenes as electrodes

Device structure	*J*_sc__(_mA cm^−2^)	*V*_oc_ (V)	FF (%)	PCE (%)	Year	References
FTO/TiO_2_/MAPbI_3_/Ti_3_C_2_T_*x*_	22.96	0.95	63.00	13.83	2019	[52]
FTO/c-TiO_2_/CPbBr_3_/Carbon:CNT:Ti_3_C_2_T_*x*_	7.16	1.357	72.97	7.09	2019	[70]
Al/PrC_60_MA/PTB7-Th: PC_71_BM/PEDOT:PSS/MXene-AgNW-PUA	14.62	0.79	61.00	7.16	2019	[71]
Al/PDINO/PC_71_BM/PBDB-T: ITIC/PEDOT:PSS/MXene-AgNW-PUA	13.98	0.86	64.00	7.70	2019	[71]
Al/PDINO/PBDB-T:ITIC:PC_71_BM/PEDOT:PSS/MXene-AgNW-PUA	14.85	0.88	63.00	8.30	2019	[71]
Glass/Ti_3_C_2_T_*x*_/PEDOT:PSS/PM6:Y6/PFN-Br/Al	24.97	0.84	64.90	13.62	2020	[72]
Ti_3_C_2_T_*x*_/n^+^-Si/n-Si/p^+^-Si/Al_2_O_3_/SiN_*x*_/Ag/Al	36.70	0.54	57.99	11.47	2019	[73]
PDMS/Au/Ti_3_C_2_T_*x*_/SiO_2_/n-Si/In:Ga	27.21	0.574	65.00	10.22	2019	[74]

**Table 4:** Summary of the key parameters for the solar cells employing MXenes as hole/electron transport layers

Device structure	*J*_sc_ (mA·cm^−2^)	*V*_oc_ (V)	FF (%)	PCE (%)	Year	References
FTO/TiO_2_/CsPbBr_3_/Ti_3_C_2_T_*x*_/Carbon (HTL)	8.54	1.444	73.08	9.01	2019	[77]
ITO/U-Ti_3_C_2_T_*x*_/PBDB-T:ITIC/Ca/Al (HTL)	15.98	0.89	64.00	9.02	2019	[53]
ITO/Ti_3_C_2_T_*x*_/PBDB-T:ITIC/PFN-Br/Al (HTL)	17.85	0.88	67.06	10.53	2019	[80]
PDMS/Au/Ti_3_C_2_T_*x*_/SiO_2_/*n*-Si/In:Ga (HTL)	27.21	0.574	65.00	10.22	2019	[74]
ITO/Ti_3_C_2_T_*x*_/CH_3_NH_3_PbI_3_/Spiro-OMeTAD/Ag (ETL)	22.63	1.08	70.00	17.17	2019	[78]
FTO/MXene/SnO_2_/perovskite/Spiro-OMeTAD/Au (ETL)	24.34	1.11	76.40	20.65	2020	[79]
ITO/UH-Ti_3_C_2_T_*x*_/PBDB-T:ITIC/MoO_3_/Al (ETL)	17.36	0.87	60.00	9.06	2019	[53]

Generally speaking, report of MXenes in application of solar cells just began since the last quarter of 2018, and the related study is still in its infant stage mainly focusing on exploration of the feasibility in varying solar cells. Device performance including PCE and stability still has plenty of room for improvement [81]. Moreover, the influence of different contaminants on Ti_3_C_2_T_*x*_ and device performance is still lacking. Here, it should also be noted that the previous reports are mainly based on experiments. Accordingly, prediction and optimization of the material properties of MXenes terminated with different functional groups based on theoretical/simulation approaches are necessary for more accurately guiding the experiments [63, 82–84].

On the other hand, the properties of MXenes including morphology, conductivity, transparency, terminating groups, WF and stability are sensitive to the fabrication process. In the meantime, considering the application scenarios, i.e., solar cells, developing the fabrication methods of MXenes with accurately controllable properties, large scale and low cost is necessary [85–87]. Moreover, besides applications in solar cells, other optoelectronic devices such as light-emitting diodes and photodetectors can find more innovations because of the unique optical, electrical and mechanical properties of MXenes. Another issue needing to be concerned is the stability of MXenes if they were exposed to air for a long time due to oxidation, which would increase their resistance and thus reduce the device performance. Thus, appropriate passivation and/or encapsulation is necessary for stable working of the related devices [88–91]. In addition, F-free synthesis of the Ti_3_C_2_T_*x*_ MXene with high purity has attracted significant attention because of the high safety and environmental friendliness.

There exist more than 100 stoichiometric MXene compositions and a limitless number of solid solutions, which would provide not only unique combinations of properties but also plenty of ways to tune them by changing the ratios of M or X elements [92]. To date, only the first discovered MXene, Ti_3_C_2_T_*x*_, has been applied in the PV field, while other types of MXenes have rarely been reported in application of solar cells. The large underexplored family of MXenes with unique properties make us believe that many exciting discoveries are to come. We optimistically expect that MXenes-based PV devices could achieve a great progress in the near future with further efforts by the researchers in this area.

Based on the above discussion and analysis, several suggestions are given for pushing exploration of MXene’s applications in solar cells: (1) deep understanding into the adjustment and optimization of the Fermi level and the electrical properties for Ti_3_C_2_T_*x*_ MXene materials terminated with varying functional groups based on theoretical prediction and experimental examination for better guiding experimental realization of high-performance solar cells; (2) further improvement of device performance such as PCE and stability based on (1), optimization of each interface in solar cells and incorporation of additional light management structures/components; (3) development of the related flexible PV devices considering the good flexibility of the MXene materials; (4) exploration of novel multifunctional integrated devices such as PV supercapacitors/secondary batteries and self-powered sensors considering the advantages of high transparency, abundant electrochemical active sites and remarkable adjustment of the electrical properties by functional groups for MXenes; and (5) in-depth study of the mechanism of the degraded performance for MXenes in air, exploration of the appropriate passivation and/or encapsulation measures and development of fabrication approaches suitable for solar cell applications. Moreover, besides the further development of the Ti_3_C_2_T_*x*_ MXene, exploring other suitable MXenes applicable in solar cells is necessary to enrich the related studies and thus to find more opportunities to realize PV devices and/or integrated devices with high performance-to-cost ratios.
